# Zoonotic Tuberculosis and Dairy Products: Comprehensive Meta‐Analysis of Prevalence and Public Health Implications

**DOI:** 10.1002/vms3.70556

**Published:** 2025-08-11

**Authors:** Sabbir Hossen Sabuz, Israt Jahan, Bristi Kona Debnath, Md. Mominul Islam, Md. Sadequl Islam

**Affiliations:** ^1^ Deprtment of Animal Science and Nutrition, Faculty of Veterinary and Animal science Hajee Mohammad Danesh Science and Technology University Dinajpur Bangladesh; ^2^ Department of Dairy and Poultry Science, Faculty of Veterinary and Animal Science Hajee Mohammad Danesh Science and Technology University Dinajpur Bangladesh; ^3^ Department of Anatomy and Histology, Faculty of Veterinary and Animal Science Hajee Mohammad Danesh Science and Technology University Dinajpur Bangladesh; ^4^ Department of Pathology Sher‐e‐Bangla Agricultural University Dhaka Bangladesh

**Keywords:** dairy products, prevalence, public health, zoonotic tuberculosis

## Abstract

**Background:**

Tuberculosis (TB) remains a global health concern, with unpasteurized milk serving as a major source of zoonotic Mycobacterium bovis transmission in endemic, low‐resource regions.

**Objective:**

This meta‐analysis investigates the prevalence of human TB cases linked to contaminated dairy products, assesses factors influencing transmission dynamics and identifies actionable strategies to reduce risks.

**Methods:**

A systematic meta‐analysis of TB cases from contaminated dairy consumption was conducted using PubMed, Web of Science and Scopus (2000–2024). Eligible studies reported human TB prevalence. Data were extracted systematically, and statistical analyses (MedCalc) assessed heterogeneity (Cochran's *Q*, *I* ^2^). Random‐ or fixed‐effects models were applied, with subgroup, sensitivity and publication bias analyses performed.

**Results:**

This meta‐analysis reviewed 25 studies from 2000 to 2024, encompassing 10,508 samples, to assess zoonotic TB linked to dairy consumption. Detection rates ranged widely from 0.77% to 49%, with molecular methods like polymerase chain reaction (PCR) showing higher sensitivity (up to 49%) than culture techniques (21%–35%). Prevalence was greater in TB‐endemic areas such as Ethiopia, Mexico and Tanzania, where raw dairy samples outperformed blood diagnostics in positivity. Notably, the highest detection rates were observed in confirmed M. bovis cases with a history of raw milk consumption, underscoring the zoonotic risk in unregulated dairy contexts. Significant heterogeneity (*I* ^2^ = 98.92%) arose from diagnostic, geographic and population differences. Pooled detection rates were 6.08% (fixed‐effects) and 22.90% (random‐effects), influenced by unpasteurized milk and poor livestock testing. Publication bias was notable (Egger's *p* = 0.0009), suggesting overrepresentation of positive small studies, highlighting challenges in standardizing zoonotic TB detection.

**Conclusion:**

Zoonotic TB from dairy products is a major concern in endemic areas due to unpasteurized milk and poor veterinary infrastructure. Enhanced pasteurization, standardized diagnostics and better surveillance are key to reducing risks, alongside global food safety standards and public health campaigns to protect vulnerable groups.

## Graphical abstract

Human *Mycobacterium bovis* detection rates varied from 0.77% to 49%, depending on diagnostic methods and regional TB prevalence. High heterogeneity (*I* ^2^ = 98.9%) reflects inconsistent testing standards and study designs. Control is challenged by COVID‐19 disruptions and resistance to pasteurization, with testing and culling remaining the most effective livestock control strategies.

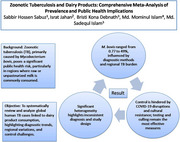

## 1 Introduction

Tuberculosis (TB) remains one of the most significant infectious diseases globally, caused predominantly by *Mycobacterium tuberculosis*, which primarily affects the lungs but can also impact other organs. A zoonotic component of TB, caused by *Mycobacterium bovis*, further complicates its epidemiology and control. The World Health Organization (WHO) estimated in 2020 that there were 10 million new TB cases and 1.4 million TB‐related deaths worldwide, underscoring its persistent public health burden (WHO 2021). Despite substantial progress in TB treatment and prevention, the disease disproportionately affects low‐ and middle‐income countries, where resource constraints hinder early diagnosis, timely treatment and robust disease control measures.

Although human‐to‐human transmission of *M. tuberculosis* remains the dominant mode of TB spread, zoonotic TB caused by *M. bovis* represents an underrecognized yet critical public health issue. *M. bovis*, a member of the *M. tuberculosis* complex, primarily affects cattle but can infect humans through zoonotic transmission pathways, such as the consumption of contaminated milk or dairy products (Gagneux 2018; Bernitz et al. 2021). Unlike *M. tuberculosis*, which spreads via respiratory droplets, *M. bovis* is transmitted to humans primarily through the ingestion of unpasteurized dairy products or, less commonly, through direct contact with infected animals (Torres‐Gonzalez et al. 2016). Populations consuming unpasteurized dairy products, particularly in low‐resource settings, are at heightened risk, exacerbating health inequities in vulnerable regions.

The socio‐economic impacts of zoonotic TB are profound, encompassing healthcare costs, lost productivity and the economic burden on smallholder farmers whose livestock may be culled to control bovine TB. These impacts are particularly acute in regions where subsistence farming is prevalent, creating a cycle of poverty and disease that undermines community resilience (Caminiti et al. 2016). Moreover, the globalization of food supply chains amplifies the risk of zoonotic TB, as unpasteurized dairy products from TB‐endemic regions may enter international markets, potentially introducing *M. bovis* into non‐endemic areas (Wilkins et al. 2008; Godwin et al. 2023). This cross‐border risk highlights the need for harmonized international food safety standards and robust trade monitoring mechanisms.

Historically, TB outbreaks were closely linked to milk consumption, particularly in Europe and North America during the 19th and early 20th centuries, when bovine TB was rampant and milk pasteurization was not yet widely practiced. Children, who were often the primary consumers of milk, were disproportionately affected due to their developing immune systems. The introduction of pasteurization and livestock vaccination programs in the 20th century significantly reduced the incidence of zoonotic TB in developed countries (Yang et al. 2024). However, in low‐ and middle‐income regions, inadequate veterinary services, weak regulatory oversight and limited access to pasteurization facilities perpetuate the risk of zoonotic TB. The public health implications of zoonotic TB extend beyond infection rates, as vulnerable populations, including children, rural communities and individuals with compromised immune systems, are disproportionately affected. These groups face heightened exposure due to unpasteurized milk consumption and limited access to healthcare (Couto et al. 2019). Addressing these disparities is crucial to reducing the global TB burden and ensuring equitable health outcomes.

This review consolidates existing evidence on the prevalence of human TB cases caused by contaminated milk or dairy products, investigates factors driving zoonotic TB transmission and proposes practical interventions to reduce risks. It specifically aims to (1) quantify the prevalence of TB cases associated with dairy consumption by analysing global data, focusing on high‐risk regions; (2) assess the impact of unpasteurized milk consumption, agricultural practices and public health measures and (3) recommend strategies to mitigate zoonotic TB risks, including enforcing pasteurization regulations, launching public education initiatives and strengthening veterinary health systems. Grounded in the One Health framework, which emphasizes the interconnectedness of human, animal and environmental health, this review seeks to address critical knowledge gaps and inform evidence‐based policies to alleviate the global burden of zoonotic TB and its public health implications. Despite the substantial public health impact of TB, there is a lack of comprehensive reviews examining human TB cases linked to contaminated milk and dairy products. This gap is particularly alarming given the ongoing threat of zoonotic TB caused by *M. bovis*, especially in low‐ and middle‐income countries where consumption of unpasteurized dairy remains widespread. The present review is vital as it aims to address this gap by synthesizing global data on TB linked to dairy consumption, identifying key risk factors and proposing actionable solutions. By adopting a One Health approach, this review not only underscores the overlooked role of dairy in TB transmission but also offers evidence‐based strategies to reduce zoonotic TB risks, ultimately contributing to lowering the global TB burden and advancing equitable health outcomes.

## 2 Methodology

This meta‐analysis systematically evaluated the prevalence of human TB cases associated with the consumption of contaminated milk or dairy products. Studies published between 2000 and 2024 were included to reflect advancements in diagnostic techniques and emerging trends in zoonotic TB transmission, aligning with contemporary public health practices. A comprehensive literature search was conducted using PubMed, Web of Science and Scopus databases. Search terms included ‘TB AND milk’, ‘*M. bovis* AND dairy products’ and ‘TB transmission AND unpasteurized milk’, combined with Boolean operators (AND, OR and NOT) to optimize retrieval of relevant studies. The search was restricted to articles published in English within the specified timeframe. Studies were included if they (1) explicitly reported human TB cases linked to milk or dairy product consumption, (2) provided clear prevalence data, (3) were conducted between 2000 and 2024 and (4) were published in peer‐reviewed journals. Exclusion criteria comprised (1) studies focusing solely on animal TB without human data, (2) studies with insufficient or ambiguous data and (3) non‐peer‐reviewed sources (e.g., conference abstracts, editorials or opinion pieces).

Data were systematically extracted from eligible studies and recorded in a standardized table. Extracted variables included prevalence rates, sample size, geographic region, study design and diagnostic methods. This process ensured consistency and facilitated comparative analysis across studies. Studies were scored based on selection, comparability and outcome criteria, ensuring reliability and validity of the findings. Statistical analyses were performed using MedCalc software (version 23.7.1). Heterogeneity among studies was assessed with Cochran's *Q* statistic and the *I* ^2^ statistic. An *I* ^2^ value >50% indicated significant heterogeneity, prompting the use of random‐effects models; otherwise, fixed‐effects models were applied. Pooled prevalence rates and 95% confidence intervals (CIs) were calculated to estimate overall zoonotic TB prevalence linked to dairy consumption. Subgroup analyses were conducted, stratifying data by geographic region, dairy product type and population demographics to identify patterns and risk factors. Sensitivity analyses tested the robustness of results by assessing the influence of individual studies on pooled estimates. Publication bias was evaluated using Egger's and Begg's tests, with *p* values <0.05 indicating significant bias.

## 3 Significance of Zoonotic TB in One Health Framework and Public Health Perspective

The One Health framework underscores the interdependence of human, animal and environmental health, recognizing that zoonotic TB, caused by M. bovis, impacts all three domains. Transmission is influenced by animal reservoirs, environmental conditions and human activities, necessitating a comprehensive approach integrating public health, veterinary science and environmental management (Couto et al. 2019). A failure to address zoonotic TB within this framework can lead to sustained outbreaks, economic burdens and challenges in global disease eradication efforts.

Animal health is critical in preventing zoonotic TB. In regions with limited veterinary infrastructure, such as Ethiopia and Tanzania, the absence of routine bovine TB testing, vaccination and culling sustains M. bovis in cattle populations, increasing human exposure through unpasteurized dairy products sold in informal markets (Fromsa et al. 2024). Strengthening livestock health programs, including regular TB testing and vaccination, can significantly reduce transmission. Environmental factors, such as milking practices and food safety regulations, also play a vital role. In India, unhygienic milking practices and inadequate pasteurization contribute to M. bovis contamination in dairy supply chains, whereas traditional dairy processing methods in Nepal have been linked to localized TB outbreaks. Ensuring food safety standards, promoting hygiene and educating the public about the risks of unpasteurized dairy products are crucial mitigation strategies (Collins et al. 2022; Kapoor et al. 2023). The global food supply chain further complicates zoonotic TB control. Dairy products from endemic regions, including Sub‐Saharan Africa and South Asia, enter international markets, posing cross‐border transmission risks (Moyo et al. 2021; Mekonnen et al. 2021). Unregulated trade of raw milk products, such as those from Mexico, exemplifies this challenge (Portillo‐Gómez et al. 2011). International collaboration to harmonize food safety standards and monitor dairy product trade is crucial in preventing the spread of M. bovis. Integrated surveillance systems improve outbreak detection and response.

Zoonotic TB disproportionately affects vulnerable populations, including children and immunocompromised individuals in low‐resource regions. In rural South Asia, children frequently consume unpasteurized milk, heightening infection risk. In Sub‐Saharan Africa, individuals with HIV/AIDS are especially vulnerable (Moyo et al. 2021; de Macedo Couto et al. 2022). Addressing these disparities requires targeted interventions, such as expanding pasteurization facilities, implementing livestock vaccination programs and increasing public awareness. Social and economic barriers also play a role in sustaining TB transmission, as many small‐scale farmers lack access to veterinary services and rely on traditional farming and dairy practices that increase exposure risks.

Historically, pasteurization significantly reduced zoonotic TB in developed countries. Before its widespread adoption, dairy products were major transmission sources, particularly among children (Yang et al. 2024). Veterinary measures, including bovine TB testing, vaccination and culling, further mitigated risk. However, in many low‐resource settings, informal dairy markets, minimal regulation and cultural preferences for raw milk sustain M. bovis transmission. Even in regions with pasteurization, gaps in heat treatment and international dairy trade can introduce contaminated products, necessitating stringent food safety standards and global cooperation. Strengthening policies that promote universal pasteurization and improving enforcement mechanisms can enhance public health outcomes (Yang et al. 2024).

New diagnostic technologies enhance TB detection and control, particularly where laboratory access is limited. Biosensors enable rapid, on‐site M. bovis detection in dairy products. Electrochemical sensors detect electrical signal changes when specific biomarkers bind to the sensor surface, offering cost‐effective, portable and rapid results (Tam et al. 2025). Surface plasmon resonance (SPR) sensors provide real‐time, label‐free detection, whereas microfluidic biosensors, or lab‐on‐a‐chip devices, allow simultaneous analysis of multiple biomarkers (Daher et al. 2023). These technologies improve food safety by identifying contamination at low concentrations. Nanomaterial‐based sensors, using gold nanoparticles, carbon nanotubes and graphene, enhance sensitivity and reliability (Seele et al. 2023; Zhu et al. 2025). Lateral flow immunoassays (LFAs) further facilitate rapid, field‐based TB detection, making them valuable in high‐risk areas with limited laboratory access (Seele et al. 2023). Additionally, advancements in whole‐genome sequencing and molecular epidemiology provide insights into strain variation, transmission dynamics and potential drug resistance patterns, enabling more effective intervention strategies. Biosensor technology revolutionizes zoonotic TB detection, complementing traditional control measures like pasteurization and veterinary monitoring (Srivastava et al. 2016). These rapid diagnostics can be deployed in informal dairy markets, border checkpoints and food safety inspections to curb M. bovis transmission. Expanding access to these innovations in resource‐limited settings will require international funding, partnerships and governmental support to scale up their implementation.

Regional case studies highlight challenges in enforcing food safety regulations in informal dairy markets. In South Asia, traditional milk consumption practices complicate TB prevention (van Der Zwan et al. 2024). Similarly, high prevalence of bovine TB has been documented in Western Asia, including recent molecular detection of M. bovis in cow's milk samples from Lorestan, Iran (Zahrakar et al. 2024), further emphasizing the zoonotic risk posed by raw milk consumption. In Sub‐Saharan Africa, poor veterinary services and high bovine TB prevalence sustain transmission (de Garine‐Wichatitsky et al. 2013). In Latin America, artisanal cheese production from raw milk remains a concern despite regulatory efforts (Andretta et al. 2019). Addressing these issues requires strengthening food safety regulations, improving veterinary infrastructure and conducting public awareness campaigns. Policies should focus on training dairy farmers in proper handling techniques, subsidizing testing programs and ensuring that regulatory bodies have adequate resources to monitor compliance. International collaboration is vital in harmonizing food safety standards, monitoring dairy product trade and sharing best practices for TB prevention. By recognizing regional challenges and prioritizing pasteurization, public health systems can mitigate zoonotic TB risks, particularly in low‐resource settings. Tailored interventions, biosensor‐based diagnostics and public health education are key to reducing TB transmission in the global dairy supply chain. Governmental policies should also incorporate financial incentives for farmers to vaccinate livestock and adopt safer dairy production methods, alongside enhancing veterinary surveillance and border control measures. However, it should be kept in mind that cattle vaccination is mainly applied in high‐prevalence regions to reduce the spread of TB within herds, and it does not directly prevent transmission to humans. Regular testing and culling of infected animals remain the most effective strategies for controlling and eradicating bovine TB.

## 4 Key Observations: Zoonotic TB Detection via Dairy Consumption

### 4.1 Factors Influencing Variability in Human TB Detection Rates Across Studies

Table 1 presents notable variability in the detection rates of human TB across different studies, regions, sample types and detection methods. It highlights that high detection rates were observed in studies conducted in countries like the United Kingdom (Jalava et al. 2007), with rates reaching up to 49% when both molecular techniques (polymerase chain reaction [PCR] and spoligotyping) and culture methods were employed (Evans et al. 2007). In contrast, studies using survey methods or simpler diagnostic techniques, such as in Tanzania (Mfinanga et al. 2003), showed lower rates (21%). Detection methods also influenced the results, with studies using advanced molecular techniques like PCR and spoligotyping generally yielding higher detection rates (up to 49%), whereas studies relying on culture methods or biochemical tests produced more moderate results (around 21%–35%). For example, studies in the United States and United Kingdom utilizing a combination of culture and molecular techniques showed a higher detection rate (up to 41%) compared to those relying on serology or basic biochemical testing (Harris et al. 2007).

**TABLE 1:** Detection of human tuberculosis linked to consumption of milk or dairy products in various study areas (2001–2023).

Study area	Total sample number	Positive sample number	Detection percentage (%)	Detection method	References
USA (San Diego, California)	51	11	21	Mantoux skin test	Besser et al. (2001)
Tanzania	38	8	21	Survey	Mfinanga et al. (2003)
USA (New York City)	4524	35	0.77	Spoligotyping and genetic deletion analysis	Winters et al. (2005)
UK	83	39	49	Culture on LJ media, biochemical tests, PCR	Jalava et al. (2007)
UK	296	41	14	molecular techniques (MIRU‐VNTR, spoligotyping)	Evans et al. (2007)
California, USA	1931	129	6.7	Culture on LJ media, biochemical tests, PCR	Harris et al. (2007)
USA	165	136	82.6	Spacer oligonucleotide typing, MIRU typing	Hlavsa et al. (2008)
Dominican Republic	400	83	20.8	Tuberculin skin test (TST) combined with chest radiographs	Cohn et al. (2009)
Ireland	6	5	83.33	Mantoux test, cultural isolation for human	Doran et al. (2009)
San Diego County, California	109	90	85	Genotyping	Rodwell et al. (2010)
Nigeria	102	6	5.88	PCR‐based detection	Waziri et al. (2010)
Mexico (Baja California)	35	18	51	PCR‐based detection	Portillo‐Gómez et al. (2011)
Argentina	39	2	5.12	Culture on LJ media, biochemical tests, PCR	Cordova et al. (2012)
Nepal	70	17	24.2	Single intradermal tuberculin test	Pandey et al. (2012)
Brazil	189	2	1.05	PCR‐based detection	Silva et al. (2013)
India	301	31	10.29	Duplex PCR	Bapat et al. (2017)
Brazil	189	2	1.05	PCR‐based detection	Silva et al. (2018)
Ethiopia	23	3	13	Comparative intradermal tuberculin test (CIDT)	Kemal et al. (2019)
South Africa	1167	70	6	Survey	Moyo et al. (2021)
Mexico (Baja California)	17	8	47	Whole genome sequencing (WGS)	Ortiz et al. (2021)
Ethiopia (Bahir Dar)	435	160	38.8	Cross‐sectional study on knowledge and practice	Hailu et al. (2021)
Ethiopia	83	34	40.96	Survey on milk consumption and zoonotic risks	Deneke et al. (2022)
Zambia	255	26	10.2	Survey and diagnostic tests (CIDT, PCR)	Monde et al. (2023)

Abbreviation: PCR, polymerase chain reaction.

Regarding sample types, studies using raw dairy samples, such as those in California (Besser et al. 2001) and New York City (Winters et al. 2005), reported relatively higher detection rates, suggesting a direct link between consumption of milk or dairy products and the likelihood of TB detection. In contrast, studies using blood samples generally showed lower detection rates, pointing to the need for more targeted sampling methods in future research. Despite this, blood samples remained significant in studies that combined them with other detection techniques (Silva et al. 2013).

### 4.2 Meta‐Analysis of Human TB Detection Rates and Study Design Influence

**FIGURE 1:**
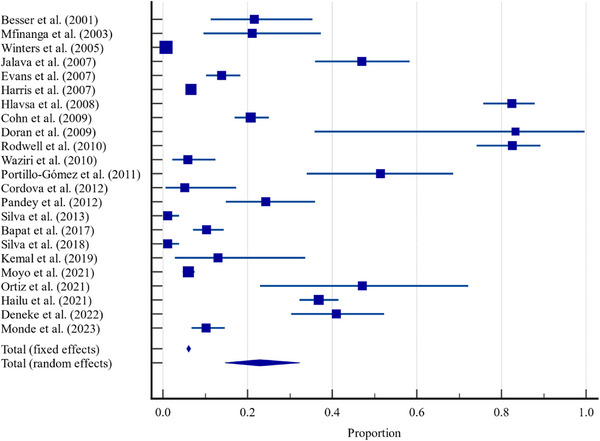
Forest plot showing the proportions of tuberculosis detection rates across studies with 95% confidence intervals. The horizontal lines represent the confidence intervals for each study, and the points indicate the estimated proportions. The dashed vertical line represents the null proportion. Studies are weighted based on their sample size, with fixed and random effects models summarized at the bottom.

**FIGURE 2:**
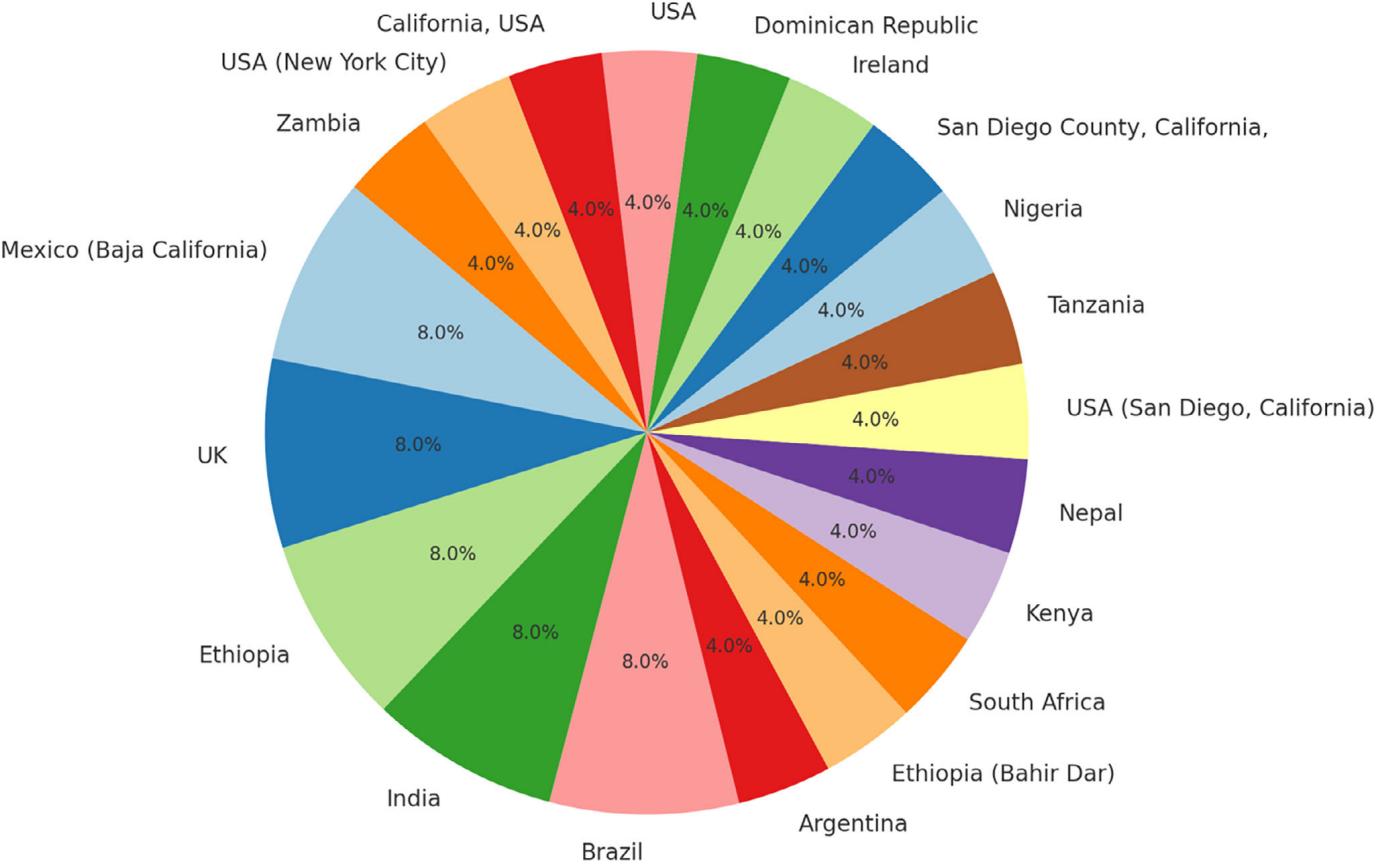
The pie chart illustrates the distribution of human tuberculosis detection rates across various countries, highlighting the varying contributions of each region to the overall detection rates. Countries like the United States, United Kingdom and Ethiopia contribute significantly, whereas others show smaller proportions.

The meta‐analysis results (Table 2) demonstrate substantial variability in the detection rates of zoonotic TB (*M. bovis*) among humans exposed to unpasteurized dairy products. Across the 23 included studies, detection rates ranged widely from as low as 0.77% (Winters et al. 2005) to as high as 49% (Jalava et al. 2007). The studies spanned diverse geographic regions, diagnostic tools, sample types and study designs. This broad variation is clearly depicted in the forest plot (Figure 1), which displays the estimated proportions from each study along with their 95% CIs (Figure 2).

**TABLE 2:** Meta‐analysis proportion for the detection rates of human tuberculosis due to consumption of milk or dairy products.

Study	Sample size	Proportion (%)	95% CI	Weight (%)	
				Fixed	Random
Besser et al. (2001)	51	21.569	11.289–35.321	0.49	4.30
Mfinanga et al. (2003)	38	21.053	9.554–37.319	0.37	4.20
Winters et al. (2005)	4524	0.774	0.539–1.074	42.97	4.62
Jalava et al. (2007)	83	46.988	35.934–58.263	0.80	4.42
Evans et al. (2007)	296	13.851	10.127–18.318	2.82	4.57
Harris et al. (2007)	1931	6.680	5.607–7.887	18.35	4.62
Hlavsa et al. (2008)	165	82.424	75.744–87.902	1.58	4.52
Cohn et al. (2009)	400	20.750	16.880–25.059	3.81	4.58
Doran et al. (2009)	6	83.333	35.877–99.579	0.066	2.95
Rodwell et al. (2010)	109	82.569	74.125–89.167	1.04	4.47
Waziri et al. (2010)	102	5.882	2.189–12.365	0.98	4.45
Portillo‐Gómez et al. (2011)	35	51.429	33.989–68.617	0.34	4.17
Cordova et al. (2012)	39	5.128	0.627–17.324	0.38	4.21
Pandey et al. (2012)	70	24.286	14.829–36.012	0.67	4.38
Silva et al. (2013)	189	1.058	0.128–3.770	1.80	4.53
Bapat et al. (2017)	301	10.299	7.106–14.299	2.87	4.57
Silva et al. (2018)	189	1.058	0.128–3.770	1.80	4.53
Kemal et al. (2019)	23	13.043	2.775–33.589	0.23	3.97
Moyo et al. (2021)	1167	5.998	4.705–7.518	11.09	4.61
Ortiz et al. (2021)	17	47.059	22.983–72.188	0.17	3.79
Hailu et al. (2021)	435	36.782	32.239–41.506	4.14	4.58
Deneke et al. (2022)	83	40.964	30.285–52.307	0.80	4.42
Monde et al. (2023)	255	10.196	6.769–14.582	2.43	4.56
Total (fixed effects)	10,508	6.082	5.633–6.556	100.00	100.00
Total (random effects)	10,508	22.907	14.694–32.327	100.00	100.00

A key factor contributing to this heterogeneity is the diversity in diagnostic methodologies. Studies using highly sensitive molecular techniques such as PCR and spoligotyping generally reported higher detection rates. For instance, Jalava et al. (2007) reported a 49% detection rate using PCR in samples from confirmed *M. bovis* cases with raw milk exposure. Conversely, studies relying on traditional culture or serological tests typically yielded lower rates, such as the 21% detection rate reported by Mfinanga et al. (2003) using conventional methods. These discrepancies emphasize the urgent need for harmonization of diagnostic protocols to ensure consistency and reliability in zoonotic TB surveillance.

The sample sizes of the included studies also varied greatly, from 38 participants (Mfinanga et al. 2003) to 4524 (Winters et al. 2005). Naturally, larger studies contributed more weight in the pooled analysis, impacting the overall detection estimates. Using a fixed‐effects model, the pooled detection rate was calculated at 6.08%. However, due to pronounced between‐study differences, the random‐effects model, which adjusts for heterogeneity, estimated a significantly higher pooled rate of 22.90%.

Statistical tests confirmed high heterogeneity among studies: Cochran's *Q* = 2041.01 and *I* ^2^ = 98.92% (95% CI: 98.74%–99.08%), indicating that nearly all variability stems from real differences in study characteristics rather than sampling error. These differences include the population's level of exposure to unpasteurized milk, the endemicity of TB in the region and critically, the type and sensitivity of the diagnostic tools used.

In summary, the findings underscore the complexity of detecting zoonotic TB in humans and the need for standardized, high‐sensitivity diagnostic approaches, especially in studies involving high‐risk exposures like raw milk consumption. Improved harmonization across diagnostic methods would facilitate more reliable cross‐study comparisons and enhance the global understanding of *M. bovis* transmission through dairy consumption.

### 4.3 Geographic Distribution of Human TB Detection Rates by Country

The pie chart (Figure 2) above illustrates the distribution of human TB detection rates across various countries based on the provided data. It reveals that countries such as the United States (San Diego, California), United Kingdom and Ethiopia each account for 8% of the overall detection rates, whereas other countries, like Zambia, Nepal and Kenya, contribute smaller proportions, each representing 4%. This chart visually highlights the geographic variability in human TB detection, with several countries exhibiting equal shares in the data. The disparity in the distribution can be influenced by factors such as healthcare infrastructure, TB prevalence and the effectiveness of diagnostic methods employed in each country (Yang et al. 2024). Countries with more advanced healthcare systems, like the United States and the United Kingdom, may have better detection methods and higher reporting capabilities, leading to a more accurate capture of TB cases. Conversely, countries with lower healthcare resources may show more limited detection and reporting, affecting their share of the total detection rates. Additionally, variations in disease prevalence across different regions contribute to the differences in detection rates observed in the pie chart.

### 4.4 Trends in Human TB Detection Rates Over Time

**FIGURE 3:**
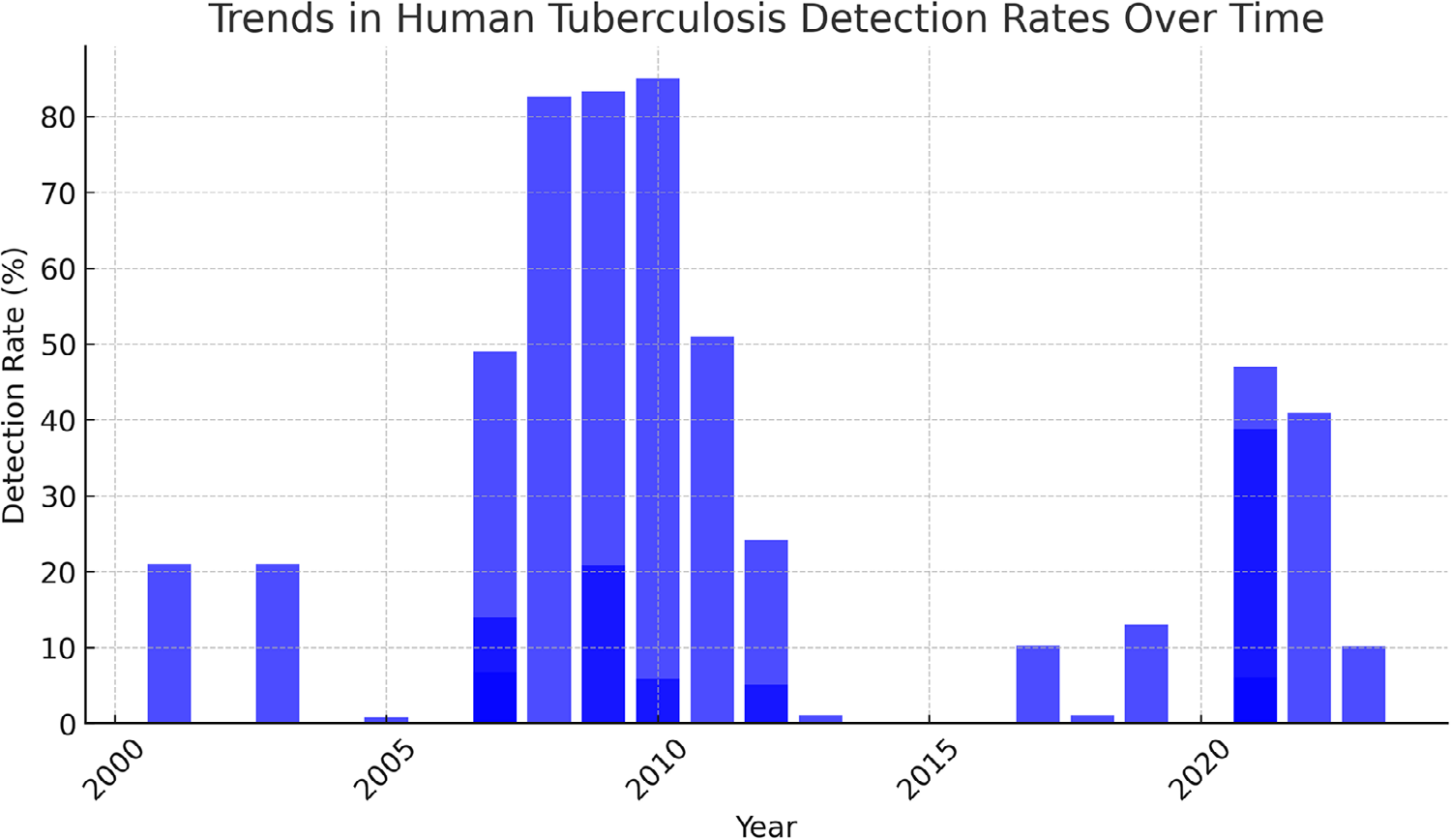
Trends in human tuberculosis detection rates over time due to milk and dairy product consumption. The bar chart highlights fluctuations in detection rates, reflecting advancements in diagnostic methods, shifts in disease prevalence and evolving public health practices.

The bar chart (Figure 3) illustrates the trends in the detection rate of human TB over time, specifically related to the consumption of milk and other dairy products. The graph shows fluctuations in detection rates, with some years experiencing higher rates, whereas others report a decline. Detection rates vary from as high as 49% to as low as 0.77%, reflecting possible changes in diagnostic methods, disease transmission and public health awareness. These trends likely reflect the evolving factors influencing TB detection, such as advancements in diagnostic techniques, shifts in disease transmission dynamics and changing public health practices and priorities in different regions (Matteelli et al. 2024). In addition to changes in diagnostic practices and public health systems, regional livestock infection rates and animal movement patterns may also influence trends in human TB detection (Olea‐Popelka et al. 2017). Areas with high bovine TB prevalence and informal dairy supply chains are particularly vulnerable to zoonotic transmission, which may contribute to temporal and geographic variations observed in the data. It is also important to note that part of the analysis period coincided with the COVID‐19 pandemic (2020–2022), during which many health systems faced disruptions (McQuaid et al. 2020). In those years, TB diagnostic and surveillance programs were often interrupted or deprioritized, likely contributing to a temporary decline in reported detection rates (e.g., the WHO reported a ∼21% drop in people receiving TB care in 2020) (WHO 2021). These pandemic‐related service disruptions could therefore explain some of the irregular fluctuations seen in the detection‐rate chart.

### 4.5 Heterogeneity in Human TB Detection Rates: Influencing Factors

The heterogeneity analysis (Table 3) reveals substantial inconsistency in TB detection rates across the included studies. The *Q* statistic (2041.01) and an *I* ^2^ value of 98.92% (95% CI: 98.74%–99.08%) indicate that nearly all the observed variation is due to systematic differences among studies, rather than random chance.

**TABLE 3:** Test for heterogeneity for the detection rates of human tuberculosis.

*Q*	2041.0126
DF	22
Significance level	*p* < 0.0001
*I* ^2^ (inconsistency)	98.92%
95% CI for *I* ^2^	98.74–99.08

Several key factors contribute to this heterogeneity:

Sample size variation: Study populations ranged from as few as 38 participants (Mfinanga et al. 2003) to over 4500 (Winters et al. 2005). Smaller studies tend to produce more variable estimates, whereas larger studies yield more stable and generalizable results.

Diagnostic methods: One of the most significant sources of variability lies in the diagnostic tools used. Studies employing advanced molecular techniques such as PCR and spoligotyping consistently reported higher detection rates. For instance, Jalava et al. (2007) recorded a 49% detection rate using PCR, whereas Mfinanga et al. (2003), using traditional culture methods, reported only 21%. In contrast, studies using older or less sensitive methods like serology or tuberculin skin tests (TSTs) generally reported lower prevalence rates.

Specimen type: The type of biological sample analysed also affected detection rates. Raw milk, sputum and tissue samples tend to offer more direct detection of *M. bovis*, whereas blood samples may yield lower sensitivity, increasing the chance of false negatives.

Geographic and regional factors: Studies conducted in high‐burden areas (e.g., Tanzania, Ethiopia) often reported higher detection rates, reflecting both increased exposure risk and possibly better targeted sampling or more intensive screening in these settings.

The combination of these variables underscores the need for harmonized diagnostic standards in zoonotic TB surveillance. Adopting consistent, high‐sensitivity diagnostic protocols across studies would greatly improve comparability and the reliability of prevalence estimates. Standardization in sample handling and test selection is essential to reduce bias and improve global assessments of zoonotic transmission risk.

Importantly, confirmed zoonotic TB cases from dairy consumption (primarily *M. bovis* in raw‐milk consumers) showed highly variable detection rates. The pooled prevalence estimates and extreme heterogeneity underscore that context, especially diagnostic method and population risk, strongly influences reported outcomes. Clear reporting of *M. bovis* cases among raw‐milk–exposed individuals is essential, as the highest detection rates in our review were found precisely in such high‐risk groups.

### 4.6 Publication Bias and Its Potential Impact on Human TB Detection Studies

The results from the publication bias tests presented in Table 4 indicate strong evidence of publication bias in the studies included in the meta‐analysis. Egger's test revealed an intercept of 8.9734 with *p* = 0.0009, suggesting the presence of publication bias, where smaller studies with positive findings are overrepresented in the literature. Additionally, Begg's test yielded a Kendall's Tau of 0.09147 with *p* = 0.5412, indicating no significant bias based on this method. The significant publication bias observed in Egger's test implies that studies with positive or extreme findings are more likely to be published, whereas studies reporting negative or inconclusive results may not be as readily published or could remain unpublished. For instance, Mfinanga et al. (2003), with a moderate detection rate of 21%, might be underrepresented in the literature compared to studies like Jalava et al. (2007), which reported much higher detection rates (49%) using molecular techniques. Several factors contribute to publication bias in TB detection studies. Selective reporting favours studies with inflated or positive results, especially those based on small sample sizes, which may exaggerate the effectiveness of diagnostic methods or detection rates due to random variation or limited statistical power. Geographic influence plays a role, as studies conducted in high‐burden regions, such as Tanzania, often report higher detection rates because of better diagnostic infrastructure and higher disease prevalence. Conversely, studies from lower burden areas may have less dramatic findings and contribute to underrepresentation in the literature. Smaller study sizes also play a part, as they are more prone to random variability, leading to inflated or extreme results, whereas larger studies typically provide more stable and accurate estimates. Smaller studies may, therefore, disproportionately impact findings in meta‐analyses. To mitigate the impact of publication bias, it is essential to promote the reporting of negative or inconclusive studies. Doing so will improve the overall reliability and validity of meta‐analyses in this field, ensuring that findings accurately reflect the true landscape of TB detection rates across different contexts and diagnostic methods.

**TABLE 4:** Publication bias test for the detection rates of human tuberculosis.

**Egger's test**	
Intercept	8.9734
95% CI	4.1443–13.8026
Significance level	*p* = 0.0009
**Begg's test**	
Kendall's Tau	0.09145
Significance level	*p* = 0.5412

## 5 Insights, Impacts and Strategies for Zoonotic TB Control

This review synthesizes existing evidence on the zoonotic transmission of TB through milk and dairy products, presenting a clear overview of the critical factors influencing transmission and the socio‐economic impacts. The review emphasizes the urgent need for integrated strategies to address the challenges posed by zoonotic TB, highlighting the role of emerging technologies, ethical considerations and the global harmonization of food safety standards.

One of the primary challenges in understanding the true burden of zoonotic TB is the variability in detection rates across different diagnostic techniques. Advanced molecular methods, such as PCR and spoligotyping, have proven to be more effective than traditional culture or serological approaches, enabling more accurate detection of the disease (Xia et al. 2024). This discrepancy highlights the need for adopting sensitive, standardized diagnostic tools, particularly in resource‐limited settings where underdiagnosis can lead to continued disease transmission. Furthermore, the importance of integrating diagnostic methods that combine diverse sample types such as raw dairy and blood samples is crucial in improving the overall epidemiological picture of zoonotic TB.

Regional disparities in the prevalence of zoonotic TB also contribute to its burden. In high‐prevalence areas, including Sub‐Saharan Africa, South Asia and Latin America, inadequate veterinary infrastructure and the absence of pasteurization practices significantly heighten the risks of zoonotic TB. Conversely, regions like North America and Europe, with well‐regulated dairy industries and stringent food safety regulations, experience lower detection rates. This disparity in burden necessitates tailored interventions that address local challenges while also adapting successful models from regions with lower prevalence. Ethical concerns surrounding strategies to control zoonotic TB, such as livestock culling and pasteurization enforcement, must be clearly addressed. Although these measures are essential for reducing TB transmission, they raise significant moral questions, particularly for rural communities that depend on livestock for their livelihoods. The enforcement of pasteurization laws, for example, can face resistance due to cultural preferences for raw milk, leading to difficulties in implementing effective public health measures. A comprehensive approach that includes compensating farmers for livestock culling and providing subsidies for pasteurization equipment can help alleviate the ethical and economic challenges associated with enforcement (de Garine‐Wichatitsky et al. 2013; Andretta et al. 2019; van Der Zwan et al. 2024).

Emerging technologies also play a critical role in controlling zoonotic TB. Digital technologies, such as mobile apps for outbreak monitoring and geospatial data analysis, offer significant potential for improving the detection, tracking and management of TB outbreaks (Ahn et al. 2021; Bag et al. 2024). These tools can enhance real‐time monitoring, inform targeted interventions and improve the efficiency of resource allocation in TB prevention programs. Integrating such technologies into public health frameworks, particularly in high‐burden regions, could revolutionize TB control efforts and help bridge gaps in diagnostics, monitoring and intervention delivery.

Controlling zoonotic TB through livestock testing, vaccination and culling poses significant challenges in low‐resource settings. Smallholder farmers in developing regions often rely heavily on their animals for both income and food security, making the loss of livestock through culling economically devastating. Without compensation schemes or alternative livelihood support, compulsory removal of infected animals is often not feasible or enforceable. Cultural practices further complicate TB prevention. In many communities, there is a strong preference for consuming raw, unpasteurized milk due to perceived health benefits or traditional beliefs. Mandating pasteurization or enforcing stricter dairy regulations without offering viable alternatives can lead to public resistance, non‐compliance and erosion of trust in public health interventions. These realities underscore that technical solutions alone are inadequate. Effective TB control strategies must be adapted to local contexts and include socio‐economic support. This may involve financial incentives such as subsidies for pasteurization equipment, compensation for culled livestock and investments in safe dairy infrastructure. Culturally sensitive education campaigns are equally important to promote behavioural change, especially in rural areas where traditional dairy practices are deeply rooted. Additionally, the limited capacity of veterinary and diagnostic services in endemic regions hampers early detection and control. Many areas lack routine testing infrastructure or trained personnel, making comprehensive surveillance and response systems difficult to implement. Strengthening local veterinary networks and investing in diagnostic capabilities should be prioritized alongside public health initiatives.

The economic and social burden of zoonotic TB is profound. Direct healthcare costs for TB diagnosis and treatment particularly for multidrug‐resistant strains strain already limited health systems. Indirect costs, including productivity loss, livestock mortality and trade restrictions, contribute to long‐term economic hardship and food insecurity. Vulnerable groups such as children, rural households and marginalized populations bear the brunt of these impacts (Atkins et al. 2022; Yang et al. 2024). Zoonotic TB also carries broader societal implications. The disease disrupts education by straining household incomes and deepens cycles of poverty in affected areas. Moreover, international trade in dairy products can be constrained by TB outbreaks, further limiting economic opportunities for producers in endemic regions. Addressing these issues requires globally coordinated efforts to improve veterinary services, standardize food safety regulations and support communities through integrated health and economic policies. Strengthening the resilience of public health and food systems will not only reduce TB transmission but also contribute to broader sustainable development goals.

Mitigation strategies for zoonotic TB must be multifaceted and adaptive to local contexts. One key strategy involves subsidizing pasteurization equipment and supporting livestock vaccination programs, which can significantly reduce TB risks. In addition, compensating farmers for livestock losses due to culling can foster compliance with TB control measures (Couto et al. 2019; Konate et al. 2024). However, addressing the socio‐cultural resistance to pasteurization will require community‐specific awareness campaigns that highlight the benefits of food safety measures while being sensitive to local customs.

A global, integrated effort is needed to combat zoonotic TB. This includes improving diagnostic capabilities, harmonizing food safety standards and providing socio‐economic support to vulnerable populations. The role of digital technologies in monitoring outbreaks and the need for ethical consideration in control measures must be central to any comprehensive TB control strategy. Only through collaboration and innovation can the global burden of zoonotic TB be reduced, fostering improved health and resilience in communities most affected by this disease. Zoonotic TB remains a global health challenge, with far‐reaching public health, economic and social consequences (Bai et al. 2024). Addressing the variability and burden of zoonotic TB requires a coordinated approach that combines robust public health measures with socio‐economic support strategies. By filling knowledge gaps, addressing ethical concerns and exploring innovative technological solutions, the global burden of zoonotic TB can be significantly reduced, enhancing health outcomes and resilience in vulnerable communities.

## 6 Preventative Measures and Recommendations

Preventing zoonotic TB requires a comprehensive, integrated approach that addresses the multiple factors driving its transmission. These strategies span across farm‐level interventions, regulatory frameworks, public awareness campaigns, ethical considerations and the use of emerging technologies. It is crucial to implement these measures over various timelines: immediate, mid‐term and long‐term, to ensure effective control and prevention. Community‐level interventions and building capacity in veterinary and public health systems are essential for the success of these efforts.

In the short term, the immediate focus should be on interventions that target existing transmission cycles and ensure rapid impact. Farm‐level interventions, including regular testing of dairy herds for M. bovis using TSTs and interferon gamma assays, are vital for early detection and containment (Doyle et al. 2022). Such measures allow for the identification of infected animals before they spread the disease within herds. Complementing this, herd health management systems should be established to monitor livestock health continuously. In high‐prevalence areas, Bacillus Calmette Guerin (BCG) vaccination programs can be introduced to reduce bacterial shedding and minimize disease severity, though ongoing research is focused on developing next‐generation vaccines with higher efficacy (Roy et al. 2014).

Safe dairy production practices, particularly pasteurization, must be universally adopted as an immediate solution to eliminate M. bovis in milk. Governments should focus on scaling small‐scale pasteurization technologies, especially for informal dairy markets, where safety practices are often lacking. Public education campaigns are also critical in the short term (Ruegg et al. 2003). These campaigns should emphasize the dangers of consuming raw milk and highlight the health and nutritional benefits of pasteurization. It is important for these messages to be culturally sensitive and contextually relevant, particularly in rural and low‐resource settings where raw milk consumption is more common. Additionally, emerging technologies should be leveraged for rapid detection. Portable PCR machines and point‐of‐care diagnostic tools offer the potential for real‐time, on‐site testing, which is particularly beneficial in remote areas where traditional diagnostic methods may be inaccessible (Hong et al. 2022).

In the mid‐term, the focus should shift toward consolidating short‐term successes and strengthening the infrastructure necessary for long‐term sustainability. Expanding vaccination programs with the BCG vaccine, particularly in high‐prevalence regions, will be important for reducing the spread of zoonotic TB. However, it should be emphasized that BCG vaccination of cattle is typically used only in high‐prevalence areas and primarily reduces TB transmission within herds—it does not directly eliminate the risk of zoonotic transmission through dairy products. Therefore, routine testing of livestock (e.g., TSTs or interferon‐γ assays) and culling of infected animals remain the cornerstone of TB control in livestock populations. Strengthening regulatory frameworks is equally vital. Governments must establish policies that enforce mandatory pasteurization and promote hygienic dairy practices, while also addressing the financial challenges faced by farmers. Financial compensation schemes for livestock losses due to culling will encourage farmers to comply with TB control measures, protecting both public health and farmers’ livelihoods. Strengthening local veterinary and public health infrastructures should be a key mid‐term priority. This includes training veterinary personnel, improving access to diagnostic tools and enhancing the capacity of public health workers to monitor outbreaks and manage disease control efforts (Thoen et al. 2016). Investments in human resources and infrastructure will lay the foundation for sustained zoonotic TB prevention.

In the long term, efforts should focus on systemic change and the development of sustainable, innovative solutions. Continued research into next‐generation vaccines is necessary to provide more effective prevention tools for both animals and humans (Kim et al. 2023). The development and widespread adoption of these vaccines should be prioritized, with a focus on making them cost‐effective and accessible to farmers in high‐prevalence regions. Long‐term solutions must also include global harmonization of food safety standards. Standardizing regulations across countries will ensure consistent and effective TB control measures, such as mandatory pasteurization and animal health monitoring (Eruaga 2024). International collaborations will be crucial in sharing best practices, promoting knowledge exchange and providing technical assistance to countries with less‐developed dairy industries. Sustaining public education initiatives over the long term is another essential aspect of prevention. Awareness campaigns must continue, focusing on new generations of consumers, ensuring that safe milk practices become ingrained in public behaviour. Engagement with community leaders, healthcare workers and educational institutions will help keep these messages relevant and effective.

Finally, the integration of emerging technologies, such as blockchain for milk traceability and geospatial mapping for outbreak surveillance, will help ensure the safety of the dairy supply chain from farm to consumer (Jagadesh et al. 2023; Mehannaoui et al. 2025). These technologies will enhance outbreak management and improve the transparency and traceability of milk production and distribution. A One Health approach, integrating human, animal and environmental health efforts, will be the cornerstone of long‐term strategies. This approach encourages collaboration among public health, veterinary medicine, agriculture and environmental science, fostering coordinated and comprehensive responses to zoonotic TB.

The prevention of zoonotic TB requires a multifaceted approach that evolves over time. Immediate actions, such as diagnostics and pasteurization, need to be complemented by mid‐term measures focused on strengthening regulatory frameworks and improving infrastructure. Long‐term strategies should focus on research, international cooperation and the integration of new technologies. By prioritizing community‐based interventions, fostering equity and ensuring sustainable development, zoonotic TB can be effectively controlled, reducing its global burden and improving health outcomes for vulnerable populations.

## 7 Limitations and Future Research Directions

Although this review provides valuable insights into the role of milk and dairy products in zoonotic TB transmission, it is important to address several key limitations and outline areas for future research. These gaps in knowledge and challenges in implementation must be explored to strengthen efforts to control and prevent zoonotic TB on a global scale.

A primary limitation is the uneven geographic focus of existing studies, with much of the research concentrated in high‐burden regions like Sub‐Saharan Africa, South Asia and Latin America (Portillo‐Gómez et al. 2011; Pandey et al. 2012; Bapat et al. 2017; Moyo et al. 2021). As a result, regions with lower TB prevalence or emerging areas, such as Eastern Europe and parts of Southeast Asia, have received less attention. This imbalance in research coverage limits the broader applicability of the findings and overlooks potentially significant regional variations in the epidemiology of zoonotic TB. Furthermore, informal dairy markets, which are widespread in low‐income regions, are often excluded from studies, leading to an underestimation of the risks associated with unregulated milk production and consumption.

In addition, diagnostic inconsistencies present another challenge in current research. Although advanced diagnostic techniques like PCR and spoligotyping offer higher detection rates, their availability remains limited, particularly in resource‐poor settings (Xia et al. 2024). This disparity in diagnostic approaches complicates the ability to compare data across studies and may result in skewed prevalence estimates. One of the most pressing issues is the underdiagnosis or misclassification of zoonotic TB, especially in extrapulmonary cases where diagnostic tools are less sensitive. This lack of accurate data contributes to underreporting, exacerbating the global burden of the disease (Gopalaswamy et al. 2021; Gheryani 2024). Another limitation lies in the narrow focus of research on *M. bovis* as the primary zoonotic agent. Other mycobacterial species capable of zoonotic transmission, such as *Mycobacterium caprae* and *Mycobacterium avium*, have received considerably less attention (Shrestha et al. 2021; Papaventsis et al. 2021). This limited focus may prevent the development of broader, more comprehensive strategies to control zoonotic TB, ignoring the full range of mycobacterial species contributing to the disease's spread.

To address these limitations, future research should aim to expand both geographic and demographic coverage. There is a clear need for studies in underrepresented regions and vulnerable populations, such as smallholder farmers, children and immunocompromised individuals. This expansion would provide a more nuanced understanding of zoonotic TB dynamics in different settings. Additionally, standardizing diagnostic protocols across studies and promoting the adoption of molecular diagnostic methods and point‐of‐care tools will improve data comparability and detection accuracy, which is essential for better surveillance and disease control.

Longitudinal studies are another critical area for future research. These studies would monitor the long‐term impact of zoonotic TB interventions, such as pasteurization and vaccination campaigns, helping to assess their effectiveness and track transmission trends over time. They will also provide valuable insights into the role of informal dairy markets in zoonotic TB transmission, enabling the design of more targeted interventions for these unregulated sectors. Furthermore, advancing vaccine research is essential for improving zoonotic TB control. Although the BCG vaccine has shown some effectiveness, the development of next‐generation vaccines, such as recombinant and thermostable formulations, could address some of the limitations of the current vaccine. Research on oral vaccines aimed at wildlife reservoirs, such as badgers and deer, is essential, as these animals can be key contributors to the transmission of zoonotic TB in specific ecosystems.

Incorporating emerging technologies into zoonotic TB control is another promising avenue for future research. Digital health platforms can facilitate real‐time surveillance and outbreak response, whereas blockchain technology could ensure supply chain transparency, enhancing milk safety from farm to consumer. Geospatial mapping tools can also assist in outbreak tracking and resource allocation, improving response times and effectiveness.

An area of research that deserves further emphasis is the development of advanced diagnostic technologies, particularly biosensor‐based methods for the rapid and cost‐effective detection of TB in dairy products. Field‐based diagnostic tools that are economically feasible and easily deployable in informal markets are critical for controlling zoonotic TB in regions with limited healthcare infrastructure. Research should focus on developing point‐of‐care biosensor devices capable of detecting *M. bovis* and other mycobacterial species directly in dairy products, providing rapid, on‐site results without the need for complex laboratory equipment. The economic costs of these devices should be a key consideration, as affordability is crucial for adoption in resource‐poor settings. These biosensors, particularly those based on electrochemical, nanomaterial‐enhanced or LFA technologies, offer low‐cost, rapid and accurate alternatives to traditional molecular diagnostic techniques. Furthermore, integrating these diagnostic tools with mobile health platforms could enable real‐time reporting and surveillance, making it easier to track outbreaks and take immediate action.

Future research should also explore the integration of biosensors into surveillance systems, particularly in informal dairy markets and at borders where dairy products are imported. Ensuring that these biosensors are scalable and user‐friendly will make them accessible to local health workers and vendors, enhancing community‐level surveillance. The inclusion of biosensor‐based diagnostics in field studies can also provide valuable data on real‐world performance and their effectiveness in preventing zoonotic TB transmission in high‐risk settings. These studies should include economic evaluations to assess the cost‐effectiveness of deploying biosensors in low‐income areas compared to more traditional diagnostic methods.

Finally, a One Health approach, which incorporates human, animal and environmental health, will be crucial in designing integrated strategies for zoonotic TB control. This interdisciplinary approach will facilitate collaboration across sectors and ensure that both animal and human health are considered in the development of diagnostic and intervention strategies. The adoption of advanced biosensors within this framework could serve as a catalyst for strengthening cross‐sectoral collaborations, improving data sharing and optimizing resource allocation.

Addressing the limitations in current research and focusing on the outlined future research priorities will significantly enhance our understanding of zoonotic TB. These efforts will ultimately lead to the development of more effective, sustainable prevention and control strategies, reducing the global burden of zoonotic TB and safeguarding public health, particularly in vulnerable populations. By investing in global funding for diagnostic tool development and improving surveillance systems, these strategies will be more robust and applicable in diverse settings, from high‐prevalence areas to informal dairy markets.

## 8 Conclusion

This review underscores the pivotal role of milk and dairy products in the zoonotic transmission of TB and highlights the urgent need for robust, multifaceted interventions to mitigate associated risks. The evidence demonstrates substantial variability in TB detection rates across studies, influenced by factors such as geographic region, diagnostic methods and sample type. These findings emphasize the complexity of zoonotic TB transmission dynamics and the critical importance of tailored prevention strategies. Key measures include widespread enforcement of pasteurization practices to eliminate pathogens like *M. bovis*, enhanced livestock surveillance through routine testing and vaccination and targeted public awareness campaigns to educate communities on the risks of consuming raw milk. Strengthening food safety regulations, particularly in informal dairy markets, will also be instrumental in reducing zoonotic TB prevalence. Furthermore, ethical considerations must guide the implementation of prevention measures to ensure fairness and sustainability, particularly for vulnerable populations. Future research must address knowledge gaps in underrepresented regions, explore innovative diagnostic and vaccination technologies and examine the socio‐economic impacts of zoonotic TB to inform evidence‐based policymaking. Collaborative international efforts will be vital to mitigating zoonotic TB risks and advancing global TB control strategies. By integrating public health measures with socio‐economic support strategies, the burden of zoonotic TB can be significantly reduced, fostering improved health and resilience in vulnerable communities worldwide.

## Author Contributions

Sabbir Hossen Sabuz contributed to conceptualization, investigation, writing – review and editing. Israt Jahan was involved in conceptualization, writing – original draft, writing – review and editing. Bristi Kona Debnath contributed to data curation, methodology, writing – review and editing. Md. Mominul Islam was responsible for methodology, investigation, writing – original draft, writing – review and editing. Md. Sadequl Islam contributed to conceptualization, methodology, data curation, investigation, supervision, writing – original draft, writing – review and editing.

## Conflicts of Interest

The authors declare no conflicts of interest.

## Peer Review

The peer review history for this article is available at https://www.webofscience.com/api/gateway/wos/peer‐review/10.1002/vms3.70556.

## Data Availability

Data will be provided upon request from the corresponding author.
